# Th17 cell differentiation induced by cytopathogenic biotype BVDV-2 in bovine PBLCs

**DOI:** 10.1186/s12864-021-08194-w

**Published:** 2021-12-07

**Authors:** Yanping Li, Tingli Liu, Guoliang Chen, Liqun Wang, Aimin Guo, Zhi Li, Li Pan, Li Mao, Xuenong Luo

**Affiliations:** 1 State Key Laboratory of Veterinary Etiological Biology, Key Laboratory of Veterinary Parasitology of Gansu Province, Lanzhou Veterinary Research Institute, CAAS, Lanzhou, 730046 China; 2 Institute of Veterinary Medicine, Jiangsu Academy of Agricultural Sciences, Key Laboratory of Veterinary Biological Engineering and Technology, Ministry of Agriculture, National Center for Engineering Research of Veterinary Bio-products, Nanjing, 210014 China

**Keywords:** BVDV-2, RNA-Seq, Th17 cell differentiation, Viral replication, Immune response

## Abstract

**Background:**

Bovine viral diarrhea virus (BVDV) is a major pathogen that causes bovine viral diarrhea/mucosal disease (BVD-MD), which has become a global infectious disease due to its wide spread and the lack of effective treatment. The process of BVDV infection is complex. Once infected, host immune cells are activated and modulated. As a major immune cell, peripheral blood lymphocyte cells (PBLCs) are the primary target of BVDV. In order to further understand the mechanism of BVDV- host interaction, the expression profiles of host lymphocytes mRNAs associated with BVDV infection were investigated by transcriptomic sequencing analysis.

**Results:**

The transcriptomic sequencing analysis was performed on bovine PBLCs infected with CP BVDV-2 GS2018 after 12 h of infection. Gene expression profiling demonstrated that 1052 genes were differentially expressed in GS2018 infected PBLCs compared with the control group. Of these genes, 485 genes were up-regulated and 567 were down-regulated. The 19 differential expressed genes (DEGs) were selected for validation using quantitative real-time PCR and the results were consistent with the results of RNA-Seq. Gene ontology enrichment and KEGG pathway analysis showed that 1052 DEGs were significantly enriched in 16 pathways, including cytokine-cytokine receptor interaction, IL17, PI3K-Akt, MAPK and TNF signaling pathway. PPI network analysis showed that IL17A, IFN-γ and TNF-α interacted with various proteins and may play crucial roles in BVDV-2 infection. Of note, we confirmed that GS2018 induced Th17 cell differentiation in PBLCs and persistently increased the expression levels of IL17A. In turn, the replication of GS2018 was inhibited by IL17A.

**Conclusion:**

In this study, the transcription changes of DEGs related to host immune responses in bovine PBLCs were caused by CP BVDV-2 infection. In particular, the effector molecules IL17A of Th17 cells were significantly up-regulated, which inhibited viral replication. These results will contribute to exploration and further understanding of the host immune response mechanism and interaction between host and BVDV-2.

**Supplementary Information:**

The online version contains supplementary material available at 10.1186/s12864-021-08194-w.

## Background

Bovine viral diarrhea virus (BVDV) is important pathogen related to bovine gastrointestinal, respiratory and reproductive diseases and causes serious bovine viral diarrhea/mucosal disease (BVD-MD), which has spread worldwide [1–3]. BVDV is a single positive strand RNA virus and belongs to the genus *Pestivirus* of the *Flaviviridae* family. Infection commonly presents with immunosuppression, fever, diarrhea, gastroenteritis, erosion of digestive tract mucosa and reproductive disorders [4, 5]. More serious is that the immune dysfunction, immunosuppression and persistent infection (PI) caused by BVDV infection promotes the further spread of the virus and increasing morbidity and mortality in animals, which brings enormous economic losses to the cattle industry.

According to the 5'UTR sequence, three genotypes (BVDV-1, BVDV-2 and BVDV-3) were identified within BVDV strains. Among them, the highly pathogenic strain, BVDV-2, usually causes severe acute infection and hemorrhagic syndrome [6, 7]. There are two biotypes in BVDV, cytopathogenic biotype (CP) and non-cytopathogenic biotype (NCP) [8]. Except for the difference in pathogenicity (CP BVDV causes cell vacuolation, shedding and necrosis, but NCP BVDV does not), the genomes of CP and NCP BVDV-2 show obvious differences in the NS2/3 coding area. A number of studies have indicated that no insertions with exogenous sequence in NS2/3 coding regions have been observed in the NCP BVDV, but an insertion, deletion or RNA recombination of exogenous sequences in the NS2/3 region was present in CP BVDV [9–11]. However, the novel CP BVDV-2 GS2018 strain isolated by our research group, is without inserted sequence in NS2/3 coding region, but showed significant cytopathic effect in MDBK cells, compared with other common CP BVDV.

The mechanisms of BVDV-host interaction and pathogenesis are complex. Successful evasion of the host immune system is the basis for persistent BVDV infection. Previous studies have indicated that BVDV infection induced host peripheral blood lymphopenia, a reduction of cell proliferation activity, damaged cell function and induced apoptosis [12–18]. Other data showed that CP BVDV infection induced type I interferon production, whereas NCP BVDV did not [19], demonstrating that NCP BVDV could avoid the innate immune response. The Npro of NCP BVDV is able to block the DNA-binding activity of interferon regulatory factor 3 (IRF-3) [20, 21], and is the main cause of immunosuppression and persistent infection. Varied levels of immune response were induced by different biotypes of BVDV, although it is generally recognized that the Th1 response was stimulated by CP BVDV infection while NCP BVDV stimulated the Th2 response [22]. All strains can cause severe depletion of T lymphocytes subgroup.

It has been reported that CD4 ^+^ effector T cells differentiate into Th17 cells, which are involved in inflammatory responses and play key roles in the host defense against extracellular pathogens [23, 24]. IL17, the main effector molecule produced by Th17 cells, is a pro-inflammatory cytokine that binds with the heterodimer IL17RA/C receptor to activate NF-κB, MAPKs, C/EBPs and other common signaling pathways, which play key roles in inducing inflammatory autoimmune diseases and host defense [24]. Studies have reported that viral infection with HBV and PRRSV could lead to up-regulated host expression of IL17, affecting virus replication and disease outcome conversely [25–27]. Here, we focus on the expression levels of immune-related cytokines, especially IL17A, in bovine PBLCs infected with GS2018, which provides theoretical support for further research on the mechanism of host immune response and pathogenesis induced by BVDV infection.

## Results

### Identifying high-quality reads

In order to obtain high quality reads from the bovine PBLCs for further analysis, the cDNA sequencing library was constructed and sequenced based on the BGISEQ-500 sequencing platform. A total of 296,230,298 raw reads (NC: 147,238,728; CP: 148,991,570) were generated. After eliminating the low-quality reads, the 269,475,890 clean reads (NC: 134,012,654, CP: 135,463,236) were obtained with the Q20 > 97%, indicating high-quality sequencing data. For further analysis, the clean reads were mapped to the reference *Bos taurus* genome (GCF_000003205.7_Btau_5.0.1) using HISAT. Approximately 86.59% of the clean reads were successfully mapped to the *Bos taurus* genome and 84.32% of the clean reads were mapped uniquely to the *Bos taurus* genome (Table 1).

**Table 1:** Statistics for sequence quality control and mapped data of samples

	Raw reads	Low quality reads	Clean reads	Clean bases (Gb)	Q20 (%)	Q30 (%)	Clean reads ratio (%)	Total mapping (%)	Uniquely Mapping (%)
NC1	49,079,576	4,145,670	44,933,906	6.74	97.23	89.75	91.55	87.45	85.36
NC2	49,079,576	4,265,738	44,813,838	6.72	97.39	90.28	91.31	87.54	85.47
NC3	49,079,576	4,814,666	44,264,910	6.64	97.5	90.59	90.19	86.42	84.16
CP1	49,079,576	3,606,904	45,472,672	6.82	97.38	90.17	92.65	86.89	84.12
CP2	50,832,418	6,211,330	44,621,088	6.69	97.53	90.72	87.78	86.47	82.48
CP3	49,079,576	3,710,100	45,369,476	6.81	97.41	90.25	92.44	84.98	84.3

### Differential expressed genes analysis

**Fig. 1:**
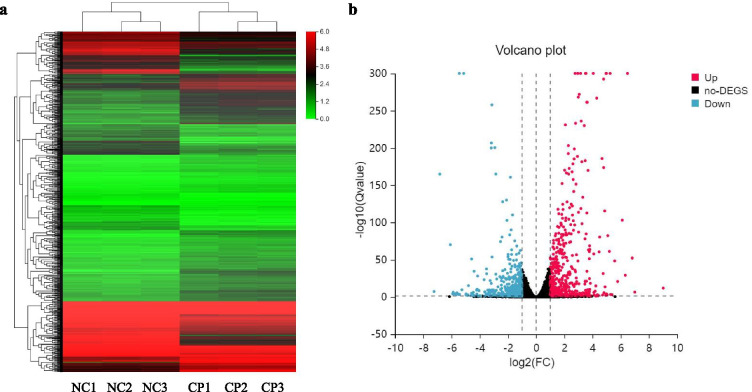
Heat map and volcano plot of the DGEs between control and BVDV-2 infected group at 12 h. **a** Heat map display the red represent the significantly up-regulated genes and the green represent the significantly down-regulated. **b** volcano plot show the red represent the significantly up-regulated genes and significantly down-regulated genes in blue

**Fig. 2:**
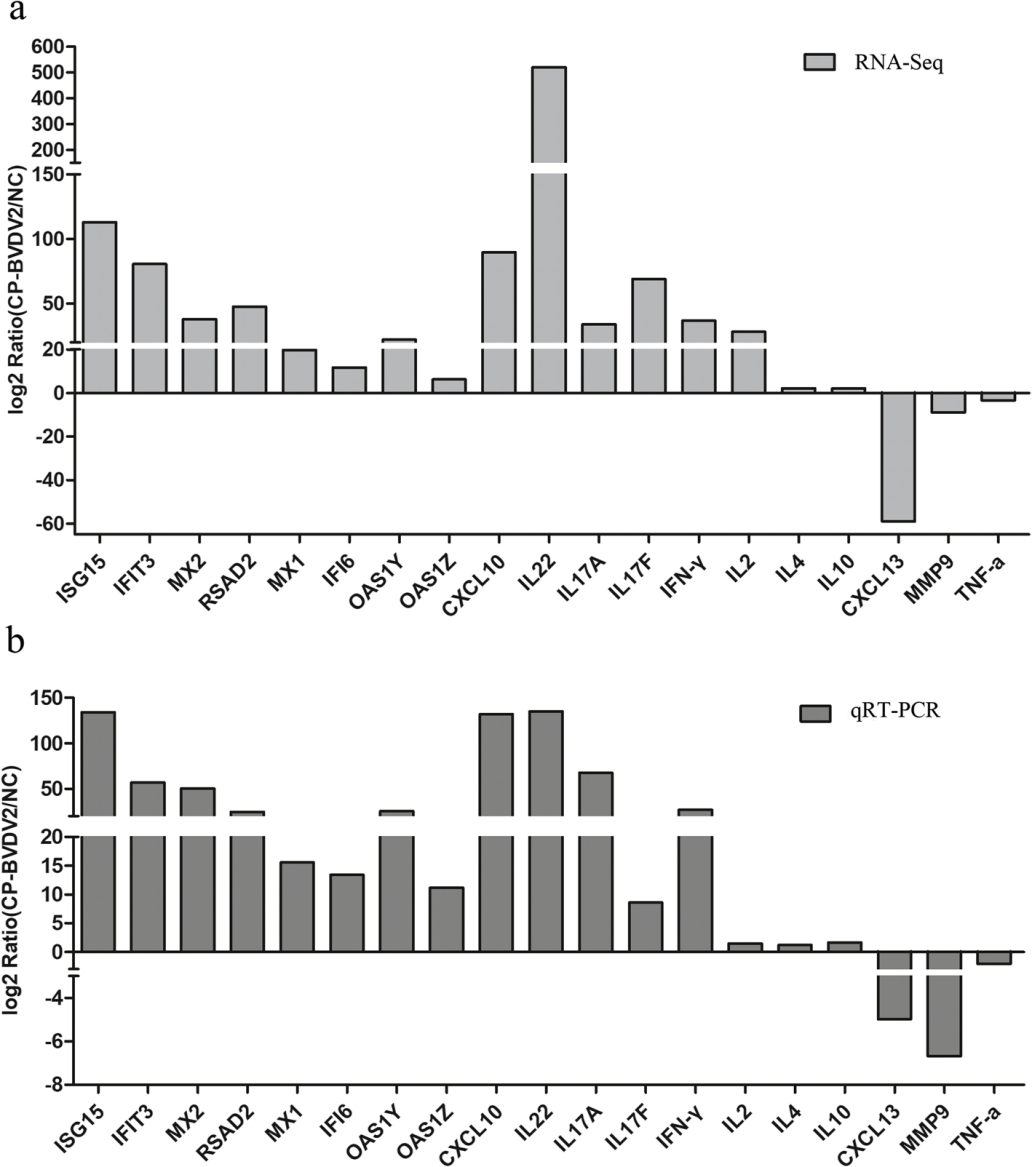
Verification of the mRNA level of some DEGs by qRT-PCR. **a** The results of RNA-Seq. **b** The results of qRT-PCR. GAPDH was used as endogenous internal reference gene and the relative content was calculated according to the 2^-ΔΔCt^ method

To investigate the mRNA expression profile of PBLCs after GS2018 infection, the DEseq2 method was used to detect the DEGs between the control (NC) and infection group (CP). As shown in Fig. 1, a total of 1052 genes were found to be significantly differentially expressed (|Log2FC| ≥ 1 and Q value < 0.05). Among them, 485 mRNAs were up-regulated and 567 mRNAs were down-regulated in the GS2018 group in comparison with the control group (Fig. 1 and Additional file 1: Tables S1 and S2). Following the preliminary analysis of the mRNA expression profile, 19 cytokine genes were selected for further qPCR validation. Accordingly, these genes presented similar expression tendency with the RNA-Seq (Fig. 2), confirming the reliability of mRNA expression profiling.

### Functional annotation and PPI analysis of DEGs

**Fig. 3:**
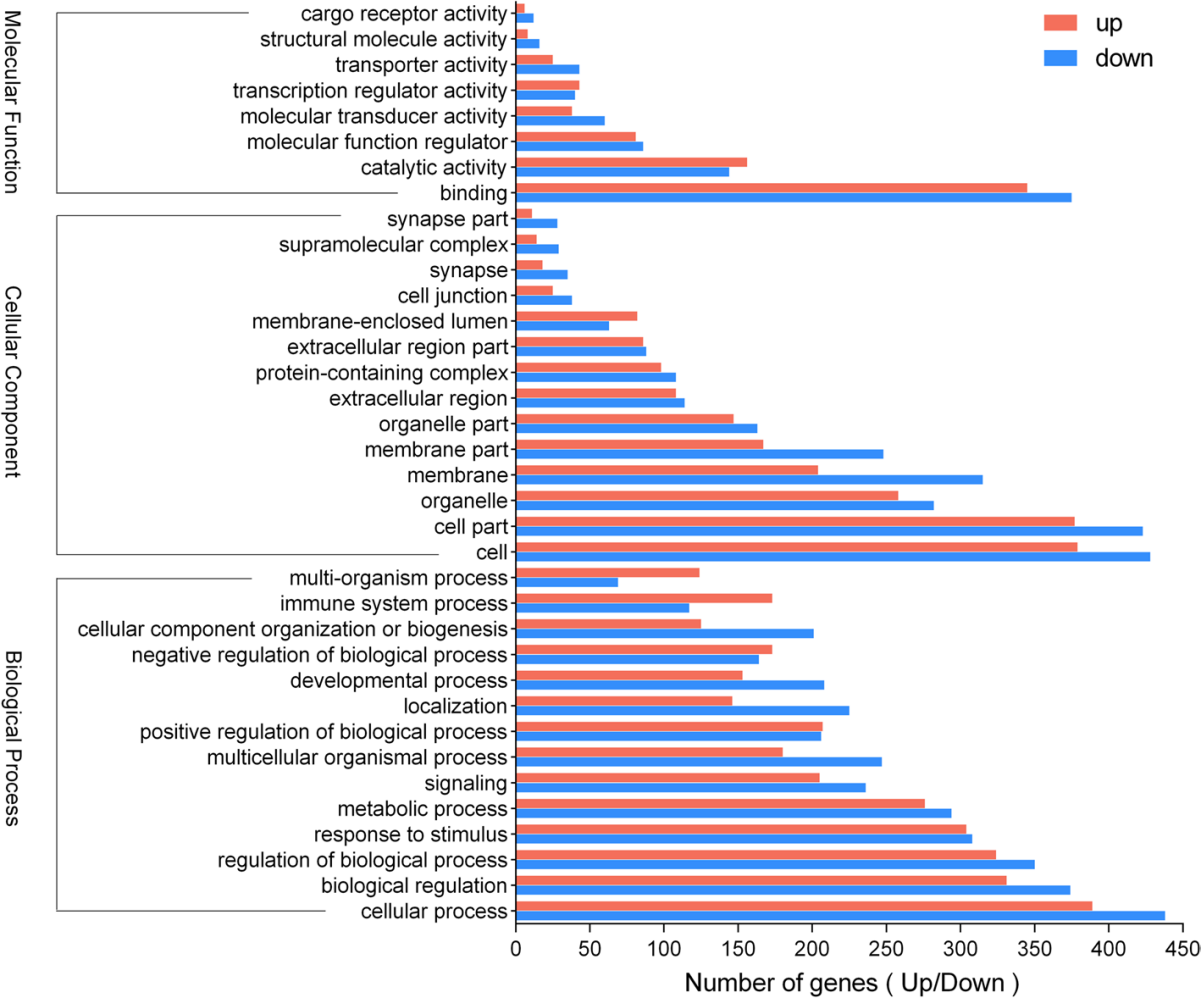
GO annotation for the DEGs between control and BVDV-2 infected bovine PBLCs. Horizontal axis represents the number of genes annotated on GO terms. Vertical axis represents the GO terms of BP, CC, and MF. Red represent the genes of up-regulated and the blue represent the down-regulated genes on different GO categories

**Fig. 4:**
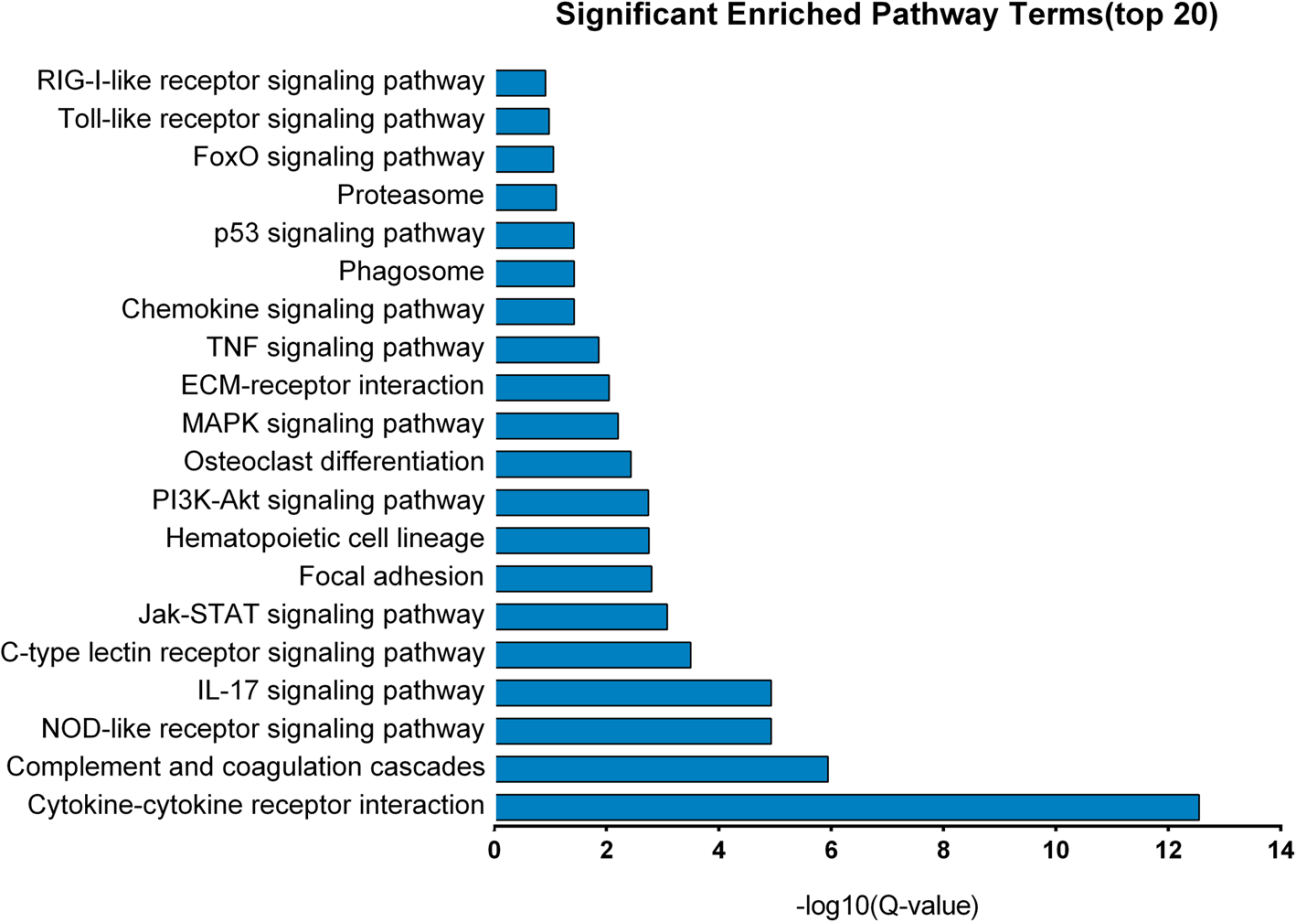
KEGG pathway enriched pathway analysis of DEGs with top 20 enrichment scores [28]

**Fig. 5:**
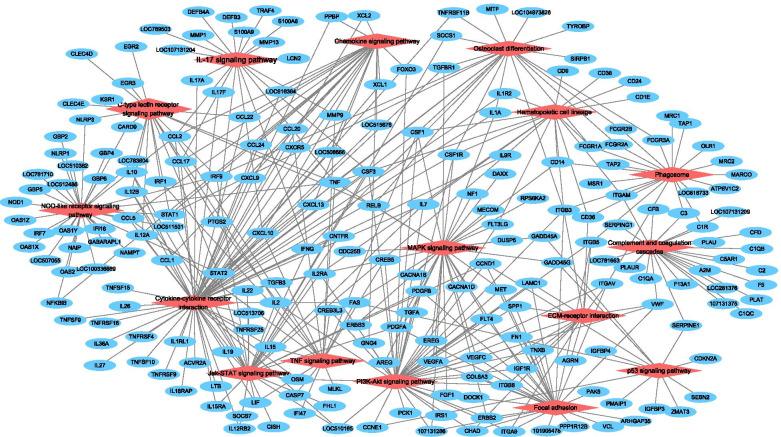
KEGG interaction network analysis. The significantly differences 16 signaling pathways were represented in red and the DEGs in these pathways were represented in blue

In order to investigate changes in the patterns and associated functions of DEGs in PBLCs infected by BVDV, GO annotation and KEGG enrichment analysis were performed on DEGs. The 1052 DEGs were annotated to 58 different GO terms, with the most annotated GO terms being cellular process (CP), biological regulation (BR), regulation of biological process (BP), response to stimulus, metabolic process (MP), signaling in BP categories, cell, cell part, organelle membrane, membrane part, organelle part in CC (cellular component) categories, and binding, catalytic activity, molecular function regulator and molecular transducer activity in MF (molecular function) categories (Fig. 3 and Additional file 2: Table S3). In addition, KEGG pathway enrichment analysis showed that these DEGs were significantly enriched in 16 pathways, including cytokine-cytokine receptor interaction, complement and coagulation cascades, NOD-like receptor, IL17, phosphatidylinositol-3-kinase (PI3K)/protein kinase B (Akt), mitogen-activated protein kinases (MAPK) and tumor necrosis factor (TNF) signaling pathway (Fig. 4 and Additional file 3: Table S4). Among these pathways, cytokine-cytokine receptor interaction was the pathway with the highest number (61) of DEGs, 46 of which were up-regulated and 15 which were down-regulated. Among these DEGs, *CXCL10*, *IL17F*, *IFN-γ*, *IL17A*, and *TNF* were also significantly enriched in the IL17 signaling pathway (Fig. 5 and Additional file 4: Table S5).

**Fig. 6:**
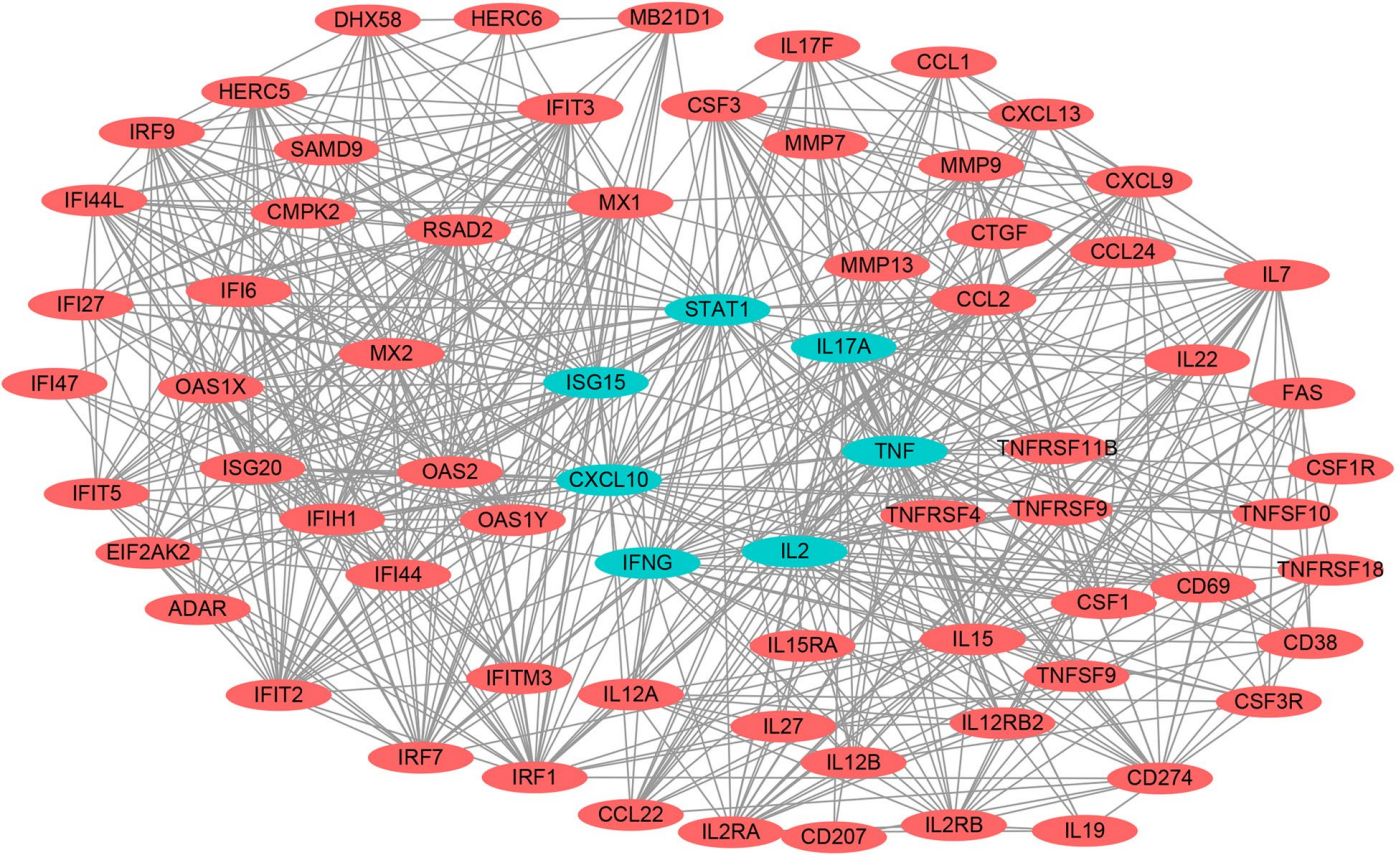
Protein-protein interaction network analysis of DEGs between control and BVDV-2 infected bovine PBLCs. Parts of DEGs interaction relationships were identified with significant values of *p* < 0.05 by using cytoscape_v3.6.1 software. The selected DEGs were represented in red circular and the green circular represents the genes with complex interaction network, the black solid line indicates the connection of each genes

Based on the data analysis results, we performed a protein-protein interaction analysis of the DEGs using the STRING database. The 4897 DEG pair interaction relationships were identified with significant values of *p* < 0.05. In order to understand better the interaction between various proteins, the candidate genes were selected for PPI network analysis using Cytoscape_v3.6.1 software (Fig. 6 and Additional file 5: Table S6). The genes involved in IFN and IL signaling pathways, including *IFN-γ*, *STAT1*, *CXCL10*, *IL17A*, *TNF-α*, *ISG15* and *IL2*, played crucial roles in this network. In addition, these DEGs were related to the biological processes of defense response to virus, innate or adaptive immune responses, and regulation of signaling receptor activity. Moreover, IL17A, IL17F and IL22 are produced by Th17 cells and were significantly up-regulated, indicating that IL17 related cytokines might play important role in innate immune response and inflammatory response during BVDV infection.

### Th17 cell differentiation was induced by GS2018 infection

**Fig. 7:**
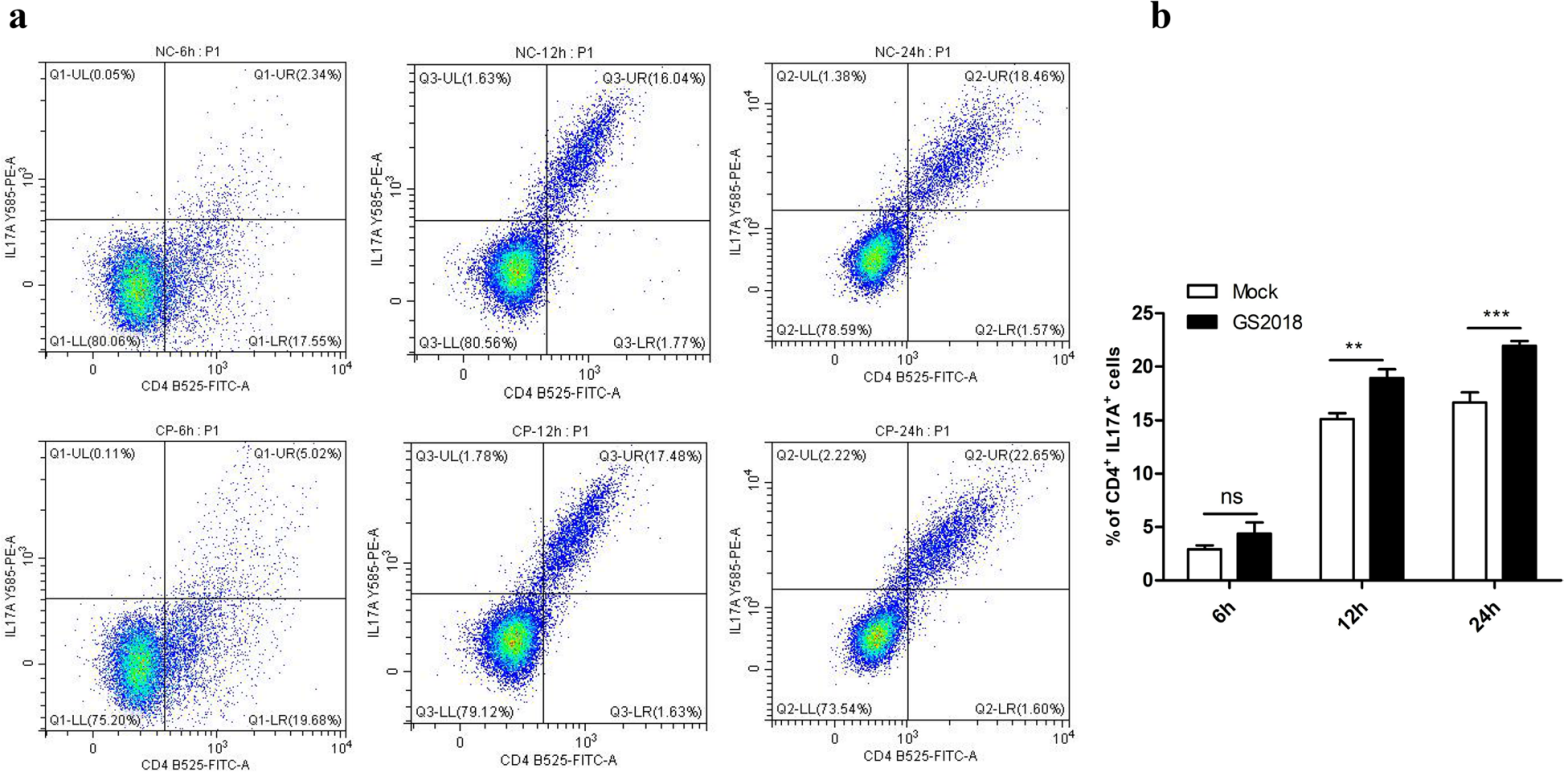
Th17 cells differentiation was detected by flow cytometry. **a** Percentages of Th17 cells labeled by the CD4 and IL17A monoclonal antibodies. **b** The bar chart shows the percentage of Th17 cells in control and GS2018 infection group at 6 h, 12 h and 24 h, respectively. Data are representative of three independent experiments and presented as means ± SDs. (ns, not significant; * *p* < 0.05; **p < 0.01)

**Fig. 8:**
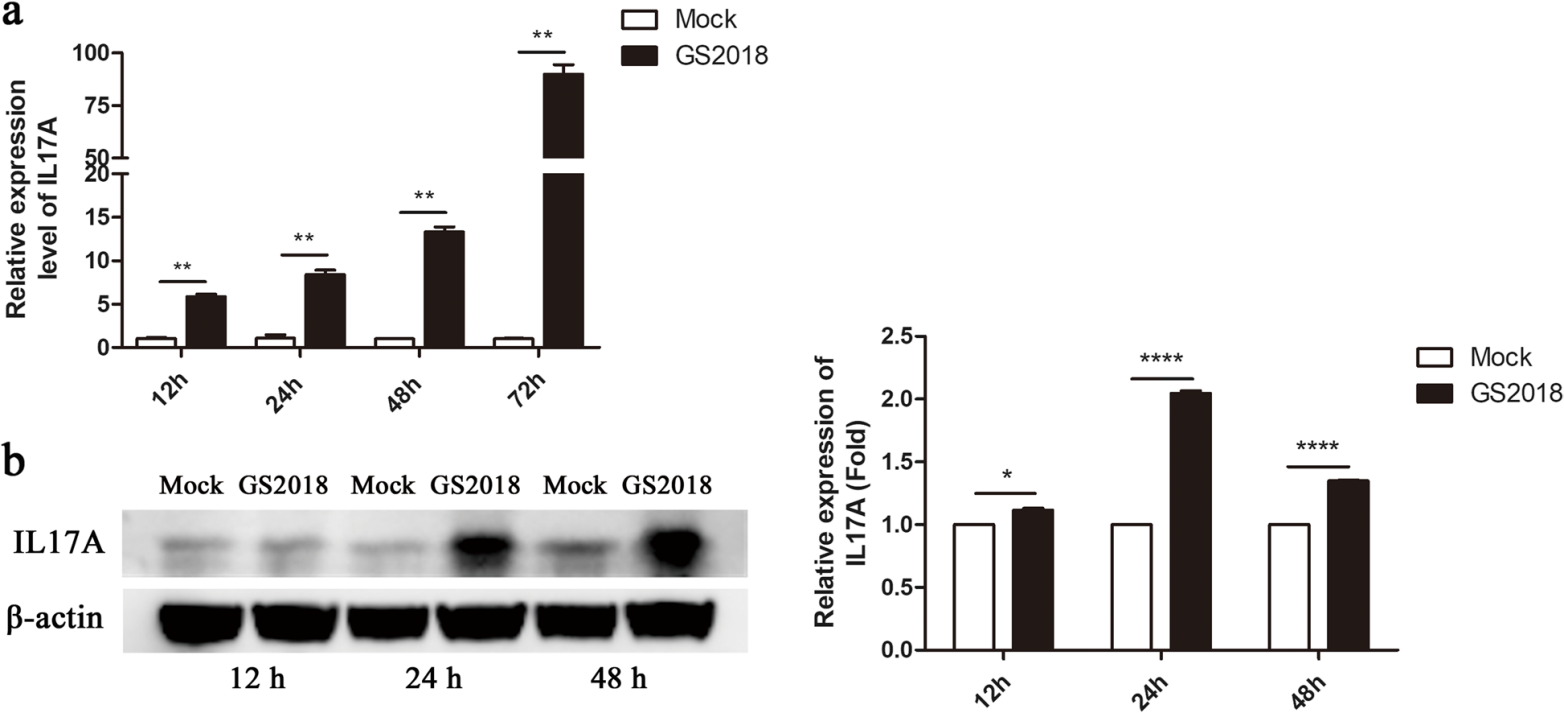
The expression of IL17A was detected by qPCR and Western blot in PBLCs. **a** qPCR detection at 12 h, 24 h, 48 h and 72 h, respectively. **b** Western blot detection at 12 h, 24 h and 48 h after GS2018 infection, respectively. The grayscale analysis of western blot result shows in bar chart. Data are representative of three independent experiments and presented as means ± SDs. (ns, not significant; * *p* < 0.05; **p < 0.01; ***p < 0.001; ****p < 0.0001)

In order to determine whether Th17 cell differentiation was induced by GS2018 infection, the percentage of Th17 cells in PBLCs was determined by flow cytometry at 6 h, 12 h and 24 h post BVDV infection (pi). Interestingly, the Th17 cells were significantly increased (22.72%; 23.27 to 23.61%, *p* < 0.01) in the GS2018 group compared with the control group (18.58%; 18.17 to 19.31%) at 24 h pi. However, no significant difference was observed between the two groups at 6 h and 12 h (*p* > 0.05) (Fig. 7). To further verify the effector molecules of Th17 cells, the expression of IL17A was detected by qPCR and western blotting in PBLCs at 12 h, 24 h, 48 h and 72 h after GS2018 infection. As shown in Fig. 8, at mRNA level, IL17A was up-regulated at least 10 folds at 48 h (*p* < 0.01) and 80 folds at 72 h (*p* < 0.01) compared with the uninfected group. The protein level of IL17A was up-regulated at 2 folds (*p* < 0.0001) at 48 h after GS2018 infection.

### IL17A inhibits GS2018 replication

**Fig. 9:**
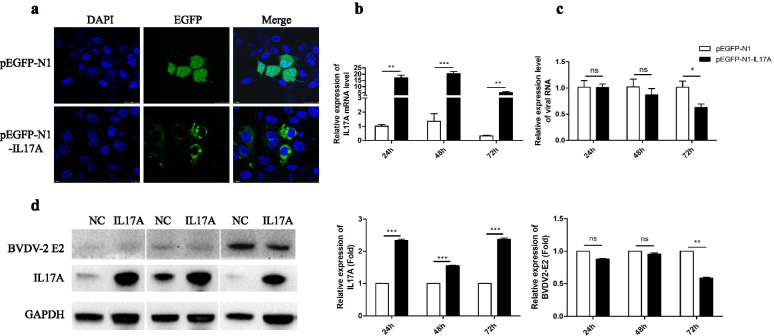
Overexpression of IL17A inhibited the replication of GS2018. The pEGFP-N1-IL17A and pEGFP-N1 (NC) fluorescence signal was detected by laser confocal microscope at 24 h (**a**). The expression of IL17A (**b**) and 5'UTR of GS2018 (**c**) were detected by qPCR. The BVDV-2 E2 (**d**) were detected by western blot. The grayscale analysis of western blot result shows in bar chart. Data are representative of three independent experiments and presented as means ± SDs. (ns, not significant; * *p* < 0.05; **p < 0.01; ***p < 0.001; ****p < 0.0001)

Based on the high expression levels of IL17A in PBLCs infected by GS2018, the PBLCs were used for a gain-of-function study. However, the transfection efficiencies of pEGFP-N1-IL17A in PBLCs were poor, so the PK15 cell line was used instead. The transfection efficiencies of pEGFP-N1-IL17A in PK15 were confirmed by fluorescence observation and qPCR (Fig. 9a-b). As expected, IL17A was localized in the cytoplasm with obvious punctate green fluorescence. We subsequently performed an overexpression assay of IL17A in PK15 cells, and then inoculated with GS2018 at a multiplicity of infection (MOI) of 1.0, which showed significant increase in IL17A (*p* < 0.01) at 24, 48 and 72 h after infection (Fig. 9b and Additional file 6: Fig. S1). Furthermore, the expression of the 5'UTR of GS2018 showed no obvious differences (*p* > 0.05) between the two groups at 24 and 48 h pi, but was down-regulated 1.6 fold at 72 h pi (*p* < 0.05, Fig. 9c). Western blotting showed that the expression level of IL17A was significantly increased in the pEGFP-N1- IL17A group at all times (Fig. 9d and Additional file 7: Fig. S2), while the expression of GS2018 E2 protein was decreased 1.7 fold at 72 h pi compared with the control group (*p* < 0.01, Fig. 9d). Taken together, these results indicated that IL17A contributed to block in vitro replication of GS2018.

## Discussion

BVD has been known for more than 70 years and is one of the most important infectious diseases in stock farming due to its serious impact on animal husbandry worldwide. While some control and treatment measures have been improved, prevention and control of the disease remains a challenge due to the host immune function incapacitation, immune suppression and persistent infection. Therefore, elucidation of the mechanisms of host immunosuppression and immune function regulation caused by BVDV is urgent.

As a high-throughput sequencing technology, RNA-Seq has been used widely in different fields, providing an effective means to study the interaction between pathogens and hosts [29–31]. Although the transcriptomes of host cells inoculated with BVDV at the early stage of infection were sequenced [32–34], the mechanism responsible for the host immune function dysregulation caused by BVDV was not clear. In addition, accumulated evidence showed that different subtypes, biotypes, and isolates of BVDV induced different levels of immune responses [35–39]. In this study, transcriptomic sequencing was performed for analysis of expression levels of genes in PBLCs at the early stage of infection with BVDV2 GS2018 strain, which provided basis data for understanding the response mechanism caused by early viral infection.

In this study, the transcriptomic analysis of bovine PBLCs indicated that most DEGs were involved in immune response, host defense, inflammatory reaction and cytokine-cytokine receptor interaction, which was consistent with previous research [32]. However, the role of the IL17 signaling pathway in the process was not frequently discussed The IFN and IL17 signaling pathway related genes, such as *IFN-γ*, *STAT1*, *ISG15*, *CXCL10*, *IL17A*, *TNF-α* and *IL2*, could interact with various cytokines, especially the significantly up-regulated interferon-stimulated genes (ISGs) and interferon-induced proteins forming the major interaction network. Previous research showed that myxovirus (influenza virus) resistance 1 (MX1) and ISG15 were up-regulated in the PBMCs from PI cattle which infected with BVDV-1 [40], suggesting that the CP and NCP BVDV could lead the production of ISGs to resist viral infection.

CD4 ^+^ T helper (Th) cells can be divided into Th1, Th2, Th17, Foxp3 (+) T-regulating cells (Tregs), Th9 and Th22 cell subsets [41], which play key roles in maintaining the balance and stability of the immune system. Under the stimulation of some cytokines, such as IL23, Th17 cells differentiate and regulate inflammation responses by producing pro-inflammatory cytokines, chemokines and recruiting neutrophil infiltration [42, 43], which was independent of lineage. Interestingly, IL17A was induced sharply by GS2018 infection in this study. Furthermore, we also found that IL22, which together with IL17A and IL17F belongs to the Th17 effector molecules, was the most differentially expressed gene, at 521 folds higher than the NC group. Generally, the production of IL17A has been recognized as the marker of Th17 cell differentiation [44–47]. In order to determine whether the high levels of pro-inflammatory cytokines were caused by Th17 cell differentiation after BVDV infection, we measured the Th17 cell differentiation by flow cytometry and the production of IL17A by qRT-PCR and western blotting. The results confirmed the increase of Th17 cell differentiation and IL17 production after GS2018 infection. In turn, the high expression of IL17A blocked the replication of GS2018, which indicated that IL17A contributed to host antiviral immunity response.

In previous studies, Th17 cell production was dependent on transcription factor retinoid-associated orphan receptor (ROR γt), which activates STAT3 under the stimulation of pro-inflammatory factors such as TGF-β, IL1β, IL-6, IL-21, and IL-23 [48–50]. However, in this study, the expression level of Th17 cell differentiation stimulating factors were not significantly changed, while its effector molecules such as IL17A, IL17F, IL22 were raised significantly. However, the mechanism of Th17 cell differentiation induced by GS2018 was still not clearly identified, and further research is needed. Recent studies confirmed that IL17 produced by Th17 cells could enhance the Th1 type immune response in mice infected by HSV-2, while in IL17-knockout mice the Th1 response was reduced and more susceptible to HSV-2 infection [51, 52]. It is worthwhile to explore the effect of IL17A production on enhancing the Th1 immune response to resist BVDV.

Furthermore, a report about HCV found that the core and NS3 proteins could promote Th17 cell differentiation through stimulation of dendritic cells to secrete a large amount of IL6 and induce the production of IL17 from CD4 ^+^ T cells, which play role in the autoimmune response of HCV patients [53]. Other research suggested that the nsp11 protein of PRRSV could up-regulate the expression of IL17A via activation of the IRAK1-PI3K-p38MAPK-C/EBP/CREB pathway, and that IL17 production was reduced and the pulmonary inflammation caused by HP-PRRSV infection was relieved by using PI3K inhibitor [26]. As discussed previously, induction of IL17 of host cells was beneficial for increasing production of pro-inflammatory cytokines, which may further promote pathogen clearance by the host. This effect was confirmed in our study, where the overexpression of IL17A significantly inhibited the replication of BVDV-2 GS2018. However, whether NS3 protein of GS2018 also has the potential to stimulate the production of IL17A remains to be investigated.

## Conclusion

In this study, the immune regulatory mechanism of bovine PBLCs was explored using RNA-seq together with qRT-PCR, western blotting and flow cytometry to evaluate the effects of Th17 cell differentiation and IL17 production induced by CP BVDV-2 GS2018 infection. We found that the replication of GS2018 was inhibited by improving the expression of IL17A, providing a new basis for the study of BVDV-host interaction. In future studies, we will focus on the molecular mechanism of Th17 cell differentiation induced by GS2018, which will provide certain theoretical support for understanding the mechanism of host immune response induced by BVDV infection.

## Materials and methods

### Cells and virus

Madin-Darby bovine kidney (MDBK, China Institute of Veterinary Drug Control, Beijing, China) were incubated in Dulbecco’s Modified Eagle’s medium-high glucose (DMEM, Hyclone Laboratories, Logan, UT, USA) containing 10% fetal bovine serum (Gibco FBS, Thermo Fisher Scientific, Waltham, MA, USA), 100 IU/ml penicillin and 100 IU/ml streptomycin at 37 °C in 5% CO_2_ humidified chamber. The CP BVDV-2 GS2018 strain (GenBank No.MN527354) was isolated from virus-contaminated commercial fetal bovine serum. The specific methodology has been described in our previous study [54].

### Virus titer assays

Purified virus particles were diluted serially (1:10) in DMEM and inoculated into a 96-well culture plate containing 100 μl/ well of MDBK cells. The eight replicates in per-dilution and one negative control group were maintained at 37 °C for 2 h. Subsequently, the supernatants were aspirated, the cells were rinsed with phosphate-buffered saline (PBS), and fresh maintenance solution (DMEM medium containing 2% FBS) was added to each well for 96 h. On the fourth day post-infection, the number of wells with CPE in each dilution was determined and the 50% the tissue culture infective dose (TCID_50_) of BVDV was calculated to be 1 × 10^6.2^ TCID_50_/100 μL by the Reed-Muench method.

### Bovine PBLCs culture and virus infection

Bovine peripheral blood was collected in EDTA anticoagulation tubes via carotid puncture. The PBLCs were separated from blood using a Bovine Peripheral Blood Lymphocyte Isolation Kit (TBD science, Tianjin, China) according to the manufacturer’s instructions. RPMI-1640 medium (Gibco, Sunnyvale, CA, USA) supplemented with 10% FBS and 1% penicillin-streptavidin was used to resuspend the PBLCs. After 4–5 h incubation at 37 °C, the PBLCs were harvested for counting and 3 × 10^6^ cells were seeded into 6-well plates. Cells in each 6-well plate were set as either the CP or NC, with MOI of 0.1 GS2018 in the CP group and the same volume RPMI-1640 medium for the NC group. 12 h pi, the three replicate samples of each group were harvested for transcriptome sequencing (Beijing Genomics Institute, Shenzhen, PR, China) and qRT-PCR analysis.

### RNA extraction, library preparation and RNA sequencing (RNA-seq)

Total RNA was extracted using TRIzol® Reagent (Invitrogen, Carlsbad, CA, USA) according to the manufacturer’s instructions. The cDNA first strand was reverse transcribed based on the polyA tail method, and we switched template at the 5′ end of the RNA and amplified the full-length cDNA by PCR. The double stranded PCR products were purified using an Agencourt AMPure XP-Medium kit (Beckman Coulter Life Sciences, San Jose, CA, USA). To obtain the single-stranded circle DNA (ssCir DNA) for library construction, the PCR products were heat denatured and circularized by the splint oligo sequence. An Agilent Technologies 2100 bioanalyzer (Agilent RNA 6000 Nano Kit, Santa Clara, CA, USA) was used to assess the integrity and concentration of the ssCir DNA, and the library was amplified to create DNA nanoballs (DNB), containing more than 300 copies of each molecule.

The DNBs were dripped into the patterned nanoarray and single-end 50 base reads were generated by combinatorial probe-anchor synthesis (cPAS) sequencing. RIN/RQN values greater than 7.0 were used for mRNA sequencing. The raw data was filtered with SOAPnuke v1.5.2 (https://github.com/BGI-flexlab/SOAPnuke) to remove reads containing sequencing adapters, low-quality base ratio (reads with a mass value below 10 that accounted for more than 20% of the total base number of the reads) and unknown bases (‘N’ base) for obtaining clean reads. The clean reads were mapped to the reference bovine genome (GCF_000003205.7_Btau_5.0.1) using HISAT2 v2.0.4 (http://www.ccb.jhu.edu/software/hisat/index.shtml). Ericscript v0.5.5 (http://ericscript.sourceforge.net/) and rMATS v3.2.5 (http://rnaseq-mats.sourceforge.net/) software were used for fusion genes and differential splicing genes (DSGs), respectively. Bowtie2 v2.2.5 (http://bowtie-bio.sourceforge.net/bowtie2) was used to align the clean reads to the gene set, which included the known and novel coding transcripts. Finally, the expression level of each gene was calculated using fragment per kilobase of exon model per million mapped reads (FPKM) by RSEM v1.2.12 (https://github.com/deweylab/RSEM).

### Differential expression genes analysis and annotation

DESeq2 with threshold of |Log2FC| ≥ 1 and Q value < 0.05 were used to identify the differentially expressed genes (DEGs). To investigate the functions of DEGs, Gene Ontology (GO) functional annotation and Kyoko Encyclopedia of Genes and Genomes (KEGG) enrichment pathway analysis were performed on all DEGs. Among them, the categories molecular function (MF), cellular component (CC) and biological process (BP) were analyzed in GO terms, with Q-value < 0.05 defined as significantly enriched terms. The KEGG Pathway analysis performed to identify the biological functions of DEGs [28], with Q-value < 0.05 considered as the significantly enriched pathways. Additionally, the protein-protein interaction network (PPI) relationship was analyzed using the String database (https://string-db.org/), and Cytoscape software was used to create the PPI network.

### Validation by qPCR

Quantitative real-time polymerase chain reaction (qPCR) was performed to verify DEGs from transcriptomic sequencing. Nineteen genes including inflammatory factors were selected for qPCR verification, including 14 up-regulated genes (*ISG15*, *IFIT3*, *MX2*, *RSAD2*, *MX1*, *IFI6*, *OAS1Y*, *OAS1Z*, *CXCL10*, *IL22*, *IL17A*, *IL17F*, *IFN-γ* and *IL2*), two genes with no differential expression (IL4 and IL10), and three down-regulated genes (*CXCL13*, *MMP9*, *TNF-α*). First, the total RNA of all samples was extracted with TRIzol reagent according to the manufacturer′s instructions. The cDNA first stand was synthesized by HiScript® III 1st Strand cDNA Synthesis Kit (+gDNA wiper). All-in-One qPCR Mix was used for qRT-PCR following the manufacturer’s instructions. The 20 μl reaction volume consisted of 10 μl 2 × all-in-one qPCR Mix, 0.4 μl Rox Reference Dye, 0.4 μl of upstream and downstream primers (10 μmol/l), 2 μl of cDNA template and 6.8 μl of RNase-free water. The primers of the selected genes were designed by Sangon Biotech (Additional file 8: Table S7). The reaction was performed using an ABI 7500 Real-Time PCR System (Thermo Fisher, Waltham, MA, USA) with the following program parameters: 95 °C for10 min, 40 cycles of 95 °C for 10 s, 60 °C for 1 min. GAPDH was used as an endogenous internal reference gene, and each assay was performed in triplicate. The relative content was calculated according to the 2^-ΔΔCt^ method.

### Recombinant plasmid pEGFP-N1-IL17A overexpression in PK15 cells

The recombinant plasmid pEGFP-N1-IL17A was constructed with *Hind* III and *Bam*H I restriction enzymes sites. The IL17 primers used in this study were as follows: sense primer, CCCAAGCTTATGGCTTCTATGAGAACTTCA, anti-sense primer, CGTGGATCCCGAGCCAAATGGCGG. Next, the pEGFP-N1-IL17A and the pEGFP-N1 vector were transfected into PK15 cells using INVI DNA RNA Transfection Reagent (Invigentech, Irvine, CA, USA) according to the manufacturer’s protocol. After 24 h, the cell nuclei were DAPI stained for 10 min at room temperature and identified by laser confocal fluorescent microscopy.

### Western blot analysis

The proteins of PBLC were extracted by using RIPA lysis buffer containing 1% protease inhibitor. After determining protein concentration, a total of 30 μl of samples were separated by 12% SDS-PAGE, transferred to a PVDF membrane and blocked with 7.5% skimmed milk at room temperature for 3 h. The membrane was cropped horizontally according to the target protein and incubated overnight with rabbit anti-IL17A polyclonal antibody (1:1000 dilution, Abcam, Pleasanton, CA, USA) and rabbit anti-BVDV E2 polyclonal antibody (1:2000 dilution, Bioss, Woburn, MA, USA) at 4 °C. Anti-mouse IgG/HRP and goat anti-rabbit IgG/HRP antibody (1**:** 4000 dilution, Beyotime Biotechnology, Beijing, China) were used as secondary antibodies. Mouse anti-GAPDH and anti-β-actin monoclonal antibody (1:2000 dilution, Beyotime Biotechnology, Beijing, China) were used as an internal standard. The membrane was reacted with ultra-sensitive ECL luminescence reagent (Beyotime Biotechnology, Beijing, China) for color detection. All experiments were performed in triplicate. Grey-scale values of each blot were measured by Image J software, and the intensity of each band was normalized to the loading control GAPDH or β-actin.

### Flow cytometry data acquisition and analysis

A total of 2 × 10^6^ PBLCs were inoculated in a 12-well plate, with CP and NC groups. Flow cytometry was performed at 6 h, 12 h and 24 h pi. Briefly, the cells were resuspended in 100 μl PBS, then filtered with 70 μm mesh screen. The cells were fixed in 4% paraformaldehyde for 30 min on ice, centrifuged and resuspended in 100 μl 1% Triton-100. The Th17 cells surface marker and IL17A were stained using FITC-labeled mouse anti-bovine CD4 (clone CC8, eBioscience, San Diego, CA, USA) antibody, PE-labeled rat anti-mouse IL17A (clone eBio17B7, eBioscience) antibody, and FITC/PE labeled mouse IgG2a kappa isotype control (clone eBM2a, eBioscience), respectively. Unstained and single-stained samples were used to adjust the fluorescence compensation level. All samples were stained on ice for 1 h, washed with PBS, and resuspended in 500 μl PBS. A flow cytometer (Beckman Coulter, Brea, CA, USA) was used to detect CD_4_^+^ and IL17A^+^ T cells and all data were analyzed using CytExpert software.

### Statistical analysis

The Student’s *t*-test was used to calculate statistically significant differences between the control and experimental groups. A *P*-value of less than 0.05 was considered to be statistically significant. All data were presented as mean ± S.D. (standard deviation). * *p* < 0.05, ** *p* < 0.01, *** *p* < 0.001 and **** *p* < 0.0001.

## Supplementary Information

**Additional file 1: Table S1.** The significantly differentially expressed mRNAs. **Table S2.** The volcano plot showed the distributions of mRNAs.

**Additional file 2: Table S3.** Go enrichment analysis of differentially expressed mRNAs.

**Additional file 3: Table S4.** KEGG pathway analysis of differentially expressed mRNAs.

**Additional file 4: Table S5.** KEGG interaction network analysis of differentially expressed mRNAs.

**Additional file 5: Table S6.** Protein-protein interaction network analysis of differentially expressed mRNAs.

**Additional file 6: Figure S1.** The expression of IL17A was detected by western blot in PBLCs at 12 h, 24 h and 48 h after GS2018 infection, respectively. The above showing original blots of the targets protein. The band of IL17A (a, b) and β-actin (c, d) were cropped horizontally from the same membrane before hybridized with the antibody and then repeated exposures by using ultra-sensitive ECL luminescence reagent. For IL17A (17 kda and 14 kda) and β-actin (42 kda), membranes were cropped at 25 kda, 35 kda and 55 kda. Thereafter each membrane was processed for respective antibody incubation and detection as described in method section.

**Additional file 7: Figure S2.** Overexpression of IL17A inhibited the replication of GS2018. The Expression of IL17A and BVDV-2 E2 were detected by western blot in PBLCs at 24 h, 48 h and 72 h after GS2018 infection, respectively. The band of IL17A (a), BVDV-2 E2 (b, c) and GAPDH (d) protein were cropped horizontally from the same membrane before hybridized with the antibody, the image below shows the marker lanes as well as the complete and clearly visible edge of the gels. For BVDV-2 E2 (42 kda), GAPDH (37 kda) and pEGFP-N1-IL17A, membranes were cropped at 36 kda, 40 kda and 60 kda. Thereafter each membrane was processed for respective antibody incubation and repeated exposures by using ultra-sensitive ECL luminescence reagent.

**Additional file 8: Table S7.** Primers used for qRT-PCR.

## Data Availability

The RNA sequencing data generated and analyzed during the present study have been submitted to NCBI project PRJNA683156 (https://www.ncbi.nlm.nih.gov/bioproject/PRJNA683156) with the accession number of SRR13318526 and SRR13318525.

## Abbreviations

BP: Biological process
BR: Biological regulation
BVD/MD: Bovine viral diarrhea/mucosal disease
BVDV: Bovine viral diarrhea virus
CC: Cellular component
CP: Cytopathogenic biotype
DEGs: Differential expressed genes
DMEM: Dulbecco’s Modified Eagle’s medium-high glucose
FBS: Fetal bovine serum
FC: Fold change
GO: Gene Ontology
KEGG: Kyoko Encyclopedia of Genes and Genomes
MDBK: Madin-Darby bovine kidney
MF: Molecular function
MOI: Multiplicity of infection
MP: Metabolic process
NCP: Non-cytopathogenic biotype
PBLCs: Peripheral blood lymphocyte cells
PBS: Phosphate buffered saline
PI: Persistent infection
PPI: Protein-protein interaction
qRT-PCR: Quantitative real-time polymerase chain reaction
RNA-seq: RNA sequencing
