# Supplementary material for: Th17 cell differentiation induced by cytopathogenic biotype BVDV-2 in bovine PBLCs
Source: BMC Genomics. 2021 Dec 7;22:884. doi: 10.1186/s12864-021-08194-w (PMC8650399; doi:10.1186/s12864-021-08194-w)
Supplement: Supplementary file 6 — Additional file 6: Figure S1. The expression of IL17A was detected by western blot in PBLCs at 12 h, 24 h and 48 h after GS2018 infection, respectively. The above showing original blots of the targets protein. The band of IL17A (a, b) and β-actin (c, d) were cropped horizontally from the same membrane before hybridized with the antibody and then repeated exposures by using ultra-sensitive ECL luminescence reagent. For IL17A (17 kda and 14 kda) and β-actin (42 kda), membranes were cropped at 25 kda, 35 kda and 55 kda. Thereafter each membrane was processed for respective antibody incubation and detection as described in method section. [file 12864_2021_8194_MOESM6_ESM.pdf]

a

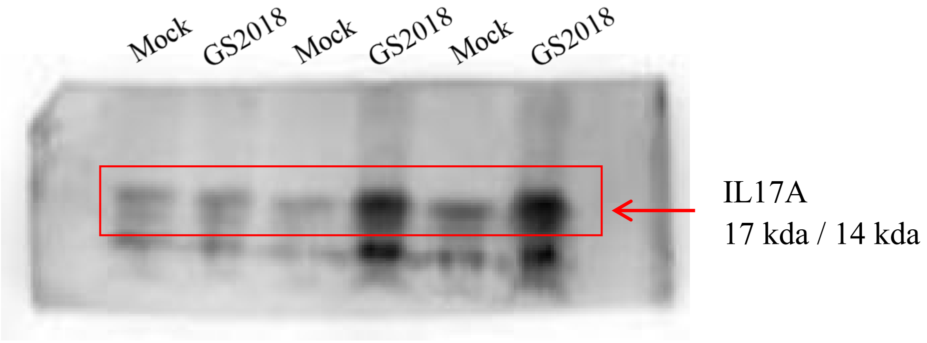

b

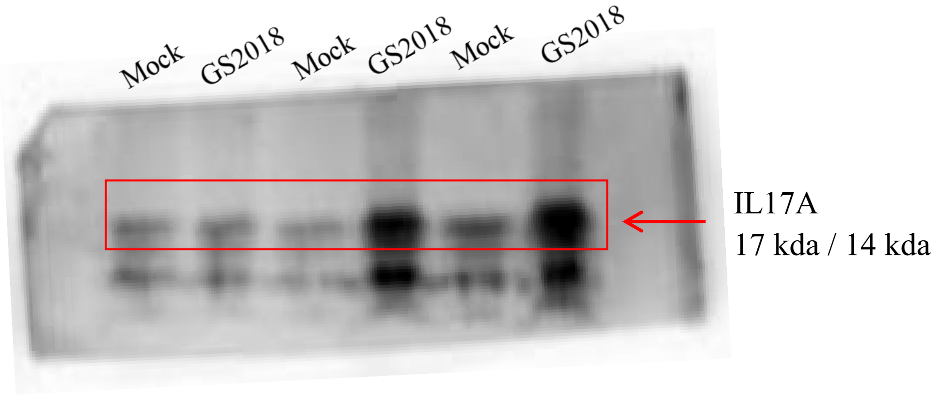

c

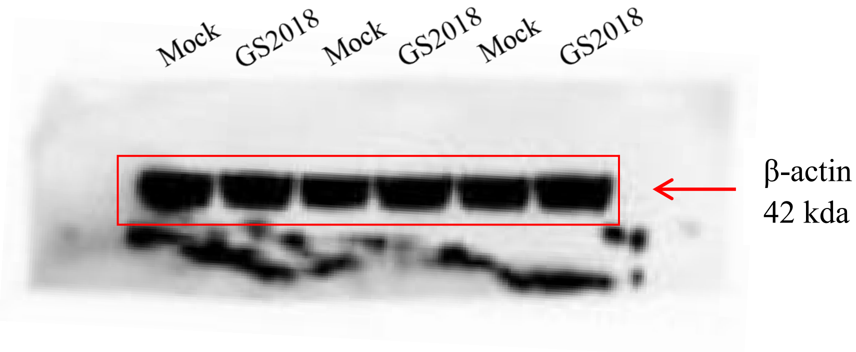

d

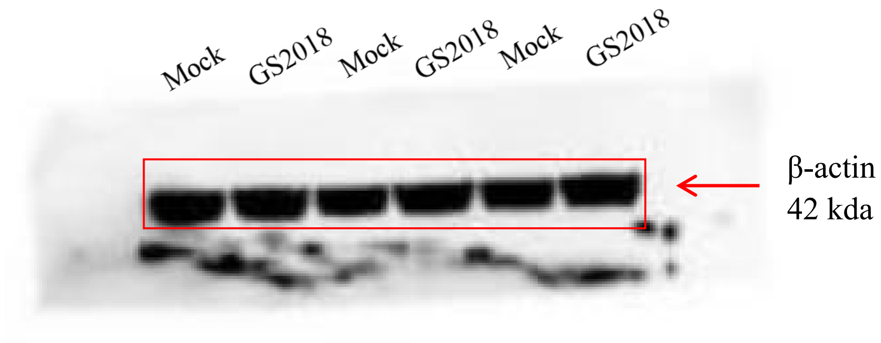

Figure S1. The expression of IL17A was detected by western blot in PBLCs at 12 h, 24 h and 48 h after GS2018 infection, respectively. The above showing original blots of the targets protein. The band of IL17A (a, b) and β-actin (c, d) were cropped horizontally from the same membrane before hybridized with the antibody

and then repeated exposures by using ultra-sensitive ECL luminescence reagent. For IL17A (17 kda and 14 kda) and β-actin (42 kda), membranes were cropped at 25 kda, 35 kda and 55 kda. Thereafter each membrane was processed for respective antibody incubation and detection as described in method section.

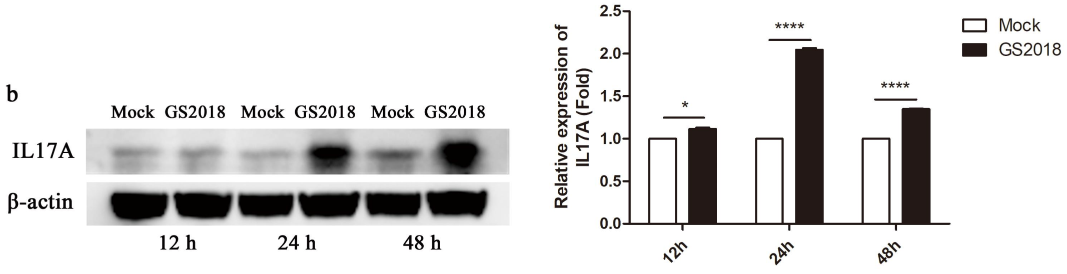

Fig. 8b The expression of IL17A was detected by western blot in PBLCs at 12 h, 24 h and 48 h after GS2018 infection, respectively. The gray analysis of western blot result shows in bar chart. Data are representative of three independent experiments and presented as means ± SDs. (ns, significant; * $p < 0.05$; ** $p < 0.01$, *** $p < 0.001$; **** $p < 0.0001$).
