# Supplementary material for: Th17 cell differentiation induced by cytopathogenic biotype BVDV-2 in bovine PBLCs
Source: BMC Genomics. 2021 Dec 7;22:884. doi: 10.1186/s12864-021-08194-w (PMC8650399; doi:10.1186/s12864-021-08194-w)
Supplement: Supplementary file 7 — Additional file 7: Figure S2. Overexpression of IL17A inhibited the replication of GS2018. The Expression of IL17A and BVDV-2 E2 were detected by western blot in PBLCs at 24 h, 48 h and 72 h after GS2018 infection, respectively. The band of IL17A (a), BVDV-2 E2 (b, c) and GAPDH (d) protein were cropped horizontally from the same membrane before hybridized with the antibody, the image below shows the marker lanes as well as the complete and clearly visible edge of the gels. For BVDV-2 E2 (42 kda), GAPDH (37 kda) and pEGFP-N1-IL17A, membranes were cropped at 36 kda, 40 kda and 60 kda. Thereafter each membrane was processed for respective antibody incubation and repeated exposures by using ultra-sensitive ECL luminescence reagent. [file 12864_2021_8194_MOESM7_ESM.pdf]

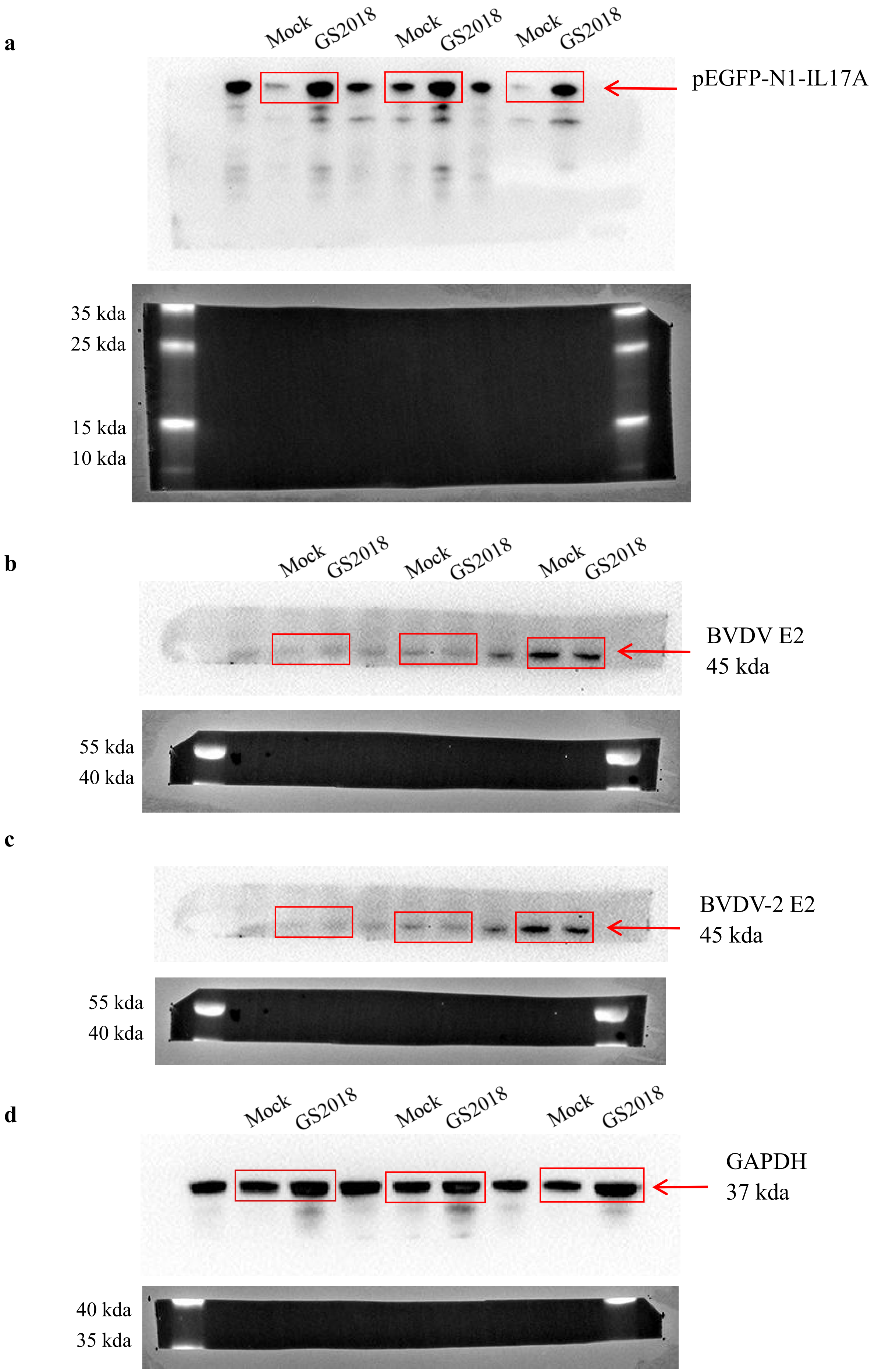
a
Mock
GS2018
Mock
GS2018
Mock
GS2018
pEGFP-N1-IL17A
35 kda
25 kda
15 kda
10 kda
b
Mock
GS2018
Mock
GS2018
Mock
GS2018
BVDV E2
45 kda
55 kda
40 kda
c
BVDV-2 E2
45 kda
55 kda
40 kda
d
Mock
GS2018
Mock
GS2018
Mock
GS2018
GAPDH
37 kda
40 kda
35 kda

Figure S2. Overexpression of IL17A inhibited the replication of GS2018. The Expression of IL17A and BVDV-2 E2 were detected by western blot in PBLCs at 24 h, 48 h and 72 h after GS2018 infection, respectively. The band of IL17A (a), BVDV-2 E2 (b, c) and GAPDH (d) protein were cropped horizontally from the same membrane before hybridized with the antibody, the image below shows the marker lanes as well as the complete and clearly visible edge of the gels. For BVDV-2 E2 (45 kda), GAPDH (37 kda) and pEGFP-N1-IL17A, membranes were cropped at 36 kda, 40 kda and 60 kda. Thereafter each membrane was processed for respective antibody incubation and repeated exposures by using ultra-sensitive ECL luminescence reagent.

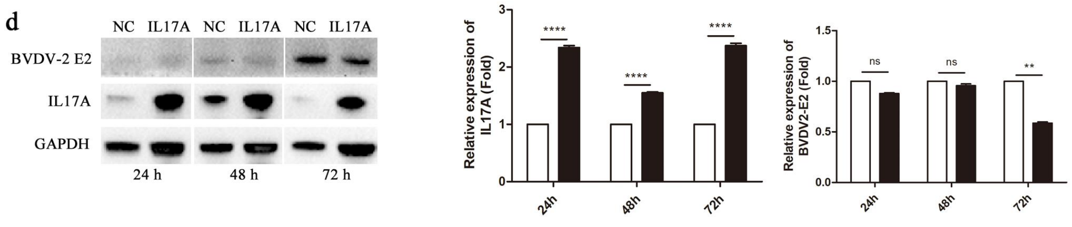

Fig.9d The BVDV-2 E2 and IL17A were detected by western blot. The gray analysis of western blot results hows in bar chart. Data are representative of three independent experiments and presented as means ± SDs. (ns, significant; *p<0.05; **p<0.01; ***p<0.001; ****p<0.0001).
