# Supplementary material for: Th17 cell differentiation induced by cytopathogenic biotype BVDV-2 in bovine PBLCs
Source: BMC Genomics. 2021 Dec 7;22:884. doi: 10.1186/s12864-021-08194-w (PMC8650399; doi:10.1186/s12864-021-08194-w)
Supplement: Supplementary file 8 — Additional file 8: Table S7. Primers used for qRT-PCR. [file 12864_2021_8194_MOESM8_ESM.pdf]

Table S7 Primers used for qRT-PCR

Gene name	GenBank	Gene ID	Fragment	Forward/Reverse Primer (5'-3')	Amplification length (bp)
ISG15	NM_174366	281871	591bp	AGAGAGCCTGGCACCAGAACC<br>AGAGAGCCTGGCACCAGAACC	104
IFI6	NM_001075588	512913	628bp	CCTCCAAGATACGGTGACAAAGCC<br>AGCAGCCGCAGGTGTAGAGTAG	100
MX1	NM_173940	280872	2434 bp	ACCGACACCAGAGACAAGAGGAAG<br>TACACGGAGGGCGGGTTCAG	106
MX2	NM_173941.2	280873	2966bp	AAGTGGAGTGGGAGATTCGGAGAG<br>GGTGATGCCAGGAAGGTCAATGAG	137
OAS1Y	NM_001040606	654488	1682bp	AAGACCACCTCCTGCCAGACG<br>TCACAACTTTGGACACCCGAACTC	129
OAS1Z	NM_001029846	519922	4746bp	CATGGGACACTGGCGAAAGGAC<br>GGTGAAGGCAAGAAGGAAGGACAG	88
RSAD2	NM_001045941	506415	2588bp	GGCAGGCTGGTGAAGTTCTGTAAG<br>ACCGTACTTCTGGAACCACCTCTC	102
IFIT3	NM_001075414	509678	2008bp	GAGAGTGGCTTGCTCACGACTG<br>GGCTGTCTGGAGAACGGAATGC	131
IL2	NM_180997	280822	798bp	ACCTCAACTCCTGCCACAATGTAC<br>TCCCCGTAGAGCTTGAAGTAGGTG	103
IL22	NM_001098379	507778	1146bp	GAGCCCTACATCTTCAACCACACC<br>CGCTTCGTCACCTGATGGATTCC	122
IL17A	NM_001008412	282863	1018bp	TCTCACAGCGAGCACAAGTTCATC<br>GCCTTCACAAGAGCCACCAGAC	120
IL17F	NM_001192082	506030	1356bp	CACAAGACGACTCAGGCACAGAAG<br>CACAATGTCAACAGCAGCAGGAAC	120
CXCL10	NM_001046551	615107	1194bp	GCAAGGGAAAGGGTGGCTCATC<br>GGAGGAAACTGTCAGTAGCAAGGC	137
IFN-γ	NM174086.1	281237	1188bp	TGATGGCATGTCAGACAGCACTTG<br>CTGAAGCGCCAGGTATAAGGTGAG	98
TNF-α	NM173966.3	280943	1712bp	CTGGCGGAGGAGGTGCTCTC<br>GGAGGAAGGAGAAGAGGCTGAGG	85
MMP9	NM_174744	282871	2350bp	GGTGCTGGCTTGCTGCTCTG<br>TTGGTGAGGTTGGTTCGTGGTTC	87
CXCL13	NM_001015576	511674	1214bp	TGAACCCTCAAGCCAAATGGACAC<br>CCACTGGAGCTGGTGAAGTTGAC	81
IL4	NM_173921	280824	576bp	ACAAATTCCTGGGCGGACTTGAC<br>TGCTCGTCTTGGCTTCATTCACAG	81
IL10	NM_174088.1	281246	745bp	GAACCACGGGCCTGACATCAAG<br>CTTCTCCACCGCCTTGCTCTTG	127
GAPDH	NM_001115114	317743	1279 bp	CGGCACAGTCAAGGCAGAGAAC<br>CCACATACTCAGCACCAGCATCAC	116
